# Effect of solvent composition on the extraction of proteins from hemp oil processing stream

**DOI:** 10.1002/jsfa.11979

**Published:** 2022-05-20

**Authors:** Eduarda M Cabral, Mahesha M Poojary, Marianne N Lund, James Curtin, Mark Fenelon, Brijesh K Tiwari

**Affiliations:** ^1^ Department of Food Chemistry and Technology Teagasc Food Research Centre Dublin Ireland; ^2^ Department of Food Science, Faculty of Science University of Copenhagen Frederiksberg C Denmark; ^3^ Department of Biomedical Sciences, Faculty of Health and Medical Sciences University of Copenhagen Copenhagen Denmark; ^4^ School of Food Science and Environmental Health, College of Sciences and Health, Technological University Dublin Dublin Ireland; ^5^ Department of Food Chemistry and Technology Teagasc Food Research Centre Co. Cork Ireland

**Keywords:** alkali extraction, salt extraction, amino acid analysis, hemp proteins, plant‐based proteins

## Abstract

**BACKGROUND:**

Hempseed meal, a by‐product of the hempseed oil processing stream, is a potential alternative source for food proteins. Efficient extraction of proteins from hempseed meal is challenging owing to differences in the structure and solubility of various protein fractions present in the seed. In the present study, protein was extracted from hempseed meal using four different solvents, including aqueous NaOH, KOH, NaHCO_3_ and NaCl, at four different concentrations with the aim of improving the recovery of protein fractions rich in essential amino acids.

**RESULTS:**

Extraction using alkaline solvents provided superior protein recovery (60–78%) compared with NaCl solution and control extractions (20–48% and 21%, respectively). The concentration of alkali or salt (0.25–1 mol L^−1^) had a minor but significant impact on the yield. Amino acid composition analysis revealed that hempseed meal contains 24% (54.5 ± 0.19 mg g^−1^) essential amino acids of total amino acids, and extraction with NaOH, KOH, NaHCO_3_ or NaCl did not improve the selective extraction of essential amino acids compared to control experiments. Sodium dodecyl sulfate–polyacrylamide gel electrophoresis (SDS‐PAGE) analysis allowed the identification of edestin and albumin in the extracts obtained with NaHCO_3_ and NaCl solvents, with results further showing that the type of extraction solvent influences protein extraction selectivity.

**CONCLUSION:**

Although alkali solvents provide superior extraction yields, extraction with water resulted in extracts containing the highest proportion of proteins bearing essential amino acids. According to the results of SDS‐PAGE, extraction using alkali solvents induced protein crosslinking. © 2022 The Authors. *Journal of The Science of Food and Agriculture* published by John Wiley & Sons Ltd on behalf of Society of Chemical Industry.

## INTRODUCTION

Hemp (*Cannabis sativa*, family Cannabinaceae) is considered to be one of the oldest and most underutilized crops growing worldwide. The crop is used to produce a variety of commercial products, such as paper, textiles, biodegradable plastics, functional foods, cosmetics and nutraceuticals.^1^ Hempseed is rich in oil, minerals and proteins, and can be used as food or food ingredient.^2^ The high demand from markets and consumers to avail sustainable, environmentally friendly and more diverse sources of proteins has transformed plant‐based proteins into an important field of research. In hemp, the cake recovered after oil extraction is a high‐value by‐product, with a considerable amount of proteins. The proteins are mainly globulins (60–80% of the total proteins) and albumins (20–40% of the total proteins).^2^, ^3^ Hemp proteins are rich in essential amino acids (EAA), with sufficient nutritional quantities for infants and/or children as recommended by FAO/WHO.^4^ On average, the whole hempseed is composed of 26–37.5% lipids, 25% crude protein and 28% fibre, while hempseed cake contains 11% lipids, 33% crude protein and 43% fibre with considerable amounts of vitamins, minerals and dietary fibre.^5^, ^6^, ^7^, ^8^

Hempseed cake is a rich source of proteins but is often discarded as a waste due to the complexity of recovering proteins. From the perspective of sustainable development and utilization of by‐products generated during food processing, hempseed cake has excellent potential for producing, for example, a valuable protein flour, meal or ingredient. Although there is literature describing the nutritional quality of hempseed and hempseed oil,^6^ relatively little information exists on the extraction and use of hempseed cake protein. To use hempseed cake as a protein source or as a protein ingredient, it is essential to achieve efficient protein recovery and separate it from unwanted fibre and residual oil. To date, most hemp protein isolates have been obtained using an adapted traditional protocol developed for soybean meal based on alkali solubilization and isoelectric precipitation.^4^ The aim of the present work was to determine optimal conditions for the extraction of protein rich in EAA from raw hemp meal cake using four different solvents – NaOH, KOH, NaHCO_3_ and NaCl – at four different concentrations. The quality of the extracted proteins was evaluated by measuring the protein extraction yield, sodium dodecyl sulfate–polyacrylamide gel electrophoresis (SDS‐PAGE) protein profile and amino acid composition.

## MATERIALS AND METHODS

### Hemp biomass and chemicals

The hempseed meal used in this study was produced as a by‐product after cold oil processing and supplied by a local Irish farm. Defatted hempseed meal pellets were ground using an electric mill (Lloytron E5012WI, Kitchen Perfected Blender with Mill, Lancshire, UK) and vacuum preserved until further analysis. Sodium hydroxide (NaOH), potassium hydroxide (KOH), sodium chloride (NaCl) and sodium bicarbonate (NaHCO_3_) were of reagent grade and purchased from Sigma‐Aldrich (St Louis, MO, USA). The amino acid calibration standard mix (analytical standard grade) was purchased from Sigma‐Aldrich (Copenhagen, Denmark). Acetonitrile (high‐performance liquid chromatography (HPLC) gradient grade) and methanol (HPLC gradient grade) were from VWR International (Søborg, Denmark). The ultrapure water used in all the experiments was obtained using a Milli‐Q system (Millipore, MA, USA).

### Proximate composition analysis

The moisture content of the dried and ground hempseed meal was determined with an infrared dryer (MA37‐1, Sartorius Laboratory Instruments GmbH & Co., Gottingen, Germany). The ash content was quantified after ignition of a weighed sample in a muffle furnace at 550 °C for 6 h according to the AOAC.942.05 method.^9^ The fat content was determined using a nuclear magnetic resonance fat analyser (Oracle, CEM Corporation, NC, USA) validated according to the AOAC 2008.06 method.^10^ Crude fibre was determined using AOAC method 962.09.^9^ The protein content of the original hempseed meal, supernatants recovered after protein extraction and residual biomasses were quantified using a nitrogen analyser (FP628, LECO Corp., MI, USA). The protein content was estimated from the nitrogen (N) content of the samples using the conversion factor 5.3 × N for all the samples.^11^

### Protein extraction

Hempseed protein isolates (HPI) were prepared from the ground hempseed meal in solution. The 16 experimental solvents were prepared by dissolving NaOH, KOH, NaCl or NaHCO_3_ in water, each at four different concentrations (0.25, 0.5, 0.75 and 1.0 mol L^−1^). Ground hempseed meal (10 g) was dispersed in each of the experimental solvents at a ratio of 1:20 (w/v) and agitated at 150 rpm using an orbital shaker (Max Q 6000 shaker incubator, Thermo Scientific, Cork, Ireland) for 24 h at room temperature. The control experiments were performed under the same conditions, but using water as the extraction solvent. The supernatants containing the soluble extracted proteins were separated from the residual biomass by filtration of each solution with a double‐layered cheesecloth with maximum pore size of 2 mm. Supernatants and residual biomasses were dried in a forced‐air circulation oven at 40 °C until constant weights were obtained (24 h) and stored at 4 °C in vacuum‐sealed bags for future analyses. The protein recovery is expressed as the percentage yield of protein extracted in the supernatant per total protein present in the crude biomass.

### Amino acid composition of extracted proteins

Dried supernatants obtained after extraction of protein from hempseed meal were subjected to total protein hydrolysis. In a typical experiment, 10 mg of sample was weighed into a 5 mL Pyrex glass vial and 3 mL of 4 mol L^−1^ methanesulfonic acid containing 0.2% (w/v) tryptamine was added. The headspace of the sample vial was flushed with nitrogen for 40 s and sealed immediately with an aluminium crimp cap with PTFE septum. The sample was hydrolysed at 110 °C for 18 h in an oven. The hydrolysate was neutralized with 4 mol L^−1^ NaOH, diluted appropriately, and mixed with an equal volume of 50 μmol L^−1^ 6‐aminocaproic acid solution (internal standard). The sample was filtered through a 0.22 μm regenerated cellulose syringe membrane filter before HPLC analysis. To determine the amino acid composition of the crude hempseed meal, the sample was directly subjected to total protein hydrolysis as described above, but without following any extraction procedure. The amino acid composition analysis was carried out on an ultra‐high‐performance liquid chromatography–fluorescence detection (UHPLC‐FLD) system (Thermo Ultimate 3000 RS, Thermo Scientific, MA, USA) equipped with an Agilent AdvanceBio AAA column (100 mm length × 3.0 mm internal diameter × 2.7 μm particle size; Agilent Technologies, CA, USA) fitted to a guard cartridge. Mobile phase A was 10 mmol L^−1^ Na_2_HPO_4_ in 10 mmol L^−1^ Na_2_B_4_O_7_ decahydrate (pH 8.2), and mobile phase B was a mixture of acetonitrile, methanol and water (45:45:10, v/v/v). A flow rate of 0.620 mL min^−1^ was applied with the following gradient program: 0–0.35 min, 2% B; 0.35–13.4 min, 57% B; 13.4–13.5 min, 100% B; 13.5–15.7 min, 100% B; 15.7–15.8 min, 2% B; 15.8–18.0 min, 2% B. Fluorescence detection was carried out by setting an excitation wavelength of 340 nm and an emission wavelength of 450 nm. The amino acids in the samples were quantified based on an internal standard calibration method using authentic amino acid calibration standards.^12^

### Sodium dodecyl sulfate–polyacrylamide gel electrophoresis

SDS‐PAGE of extracted proteins was carried out according to a method described by Jansson *et al*.,^13^ with some modifications. In a typical experiment, the extract (10 mg) was vortexed with 200 μL of 5% SDS and heated at 70 °C for 10 min to solubilize proteins. The sample was further diluted in lithium dodecyl sulfate sample buffer (Thermo Scientific, MA, USA) to obtain a protein concentration of ~1 mg mL^−1^. To reduce the reducible crosslinks present in proteins, 180 μL diluted sample was mixed with 20 μL of 1 mol L^−1^ dithiothreitol (DTT) solution (referred to as reduced samples). A control sample (referred to as a non‐reduced sample) without DTT treatment was also included by replacing DTT with an equal volume of water. Samples were heated at 70 °C for 10 min and 4 μL of sample (corresponding to ~4 μg protein) was loaded into a NuPAGE Novex 12% Bis‐tris gel. A pre‐stained protein molecular weight marker (3 μL, Novex Sharp Pre‐stained Protein Standard, Thermo Scientific, MA, USA) was also loaded into the gel. Electrophoresis was carried out at 200 V for 50 min in 3‐(*N*‐morpholino)propane sulfonic acid running buffer using an XCell SureLock Mini‐Cell electrophoresis chamber (Thermo Scientific, MA, USA).

### Statistical analysis

In this study, all measurements were carried out in duplicate. SAS (version 9.4, SAS Institute Inc., Cary, NC, USA) was used for statistical processing and analysis of data. Means and standard deviations were calculated for all analysis data. Mean differences in the recovery of protein and amino acids among the four types of solvents were analysed using general linear models and Tukey's post hoc test. Verification of significance was conducted for all statistical analyses at the level of *P* < 0.05.

## RESULTS AND DISCUSSION

### Proximate composition analysis

The proximate composition (moisture, ash, fat, protein and fibre) of the crude hempseed meal is summarized in Table 1. The hempseed meal was characterized by a high crude protein content 259.0 g kg^−1^, demonstrating its potential as a source of protein ingredient. The levels of lipids and ash were at concentrations ranging from 6.13% to 7.61% in the sample. The crude hempseed cake composition showed a similar trend to the values reported by Shen *et al*.^14^

**Table 1:** Proximate composition (moisture, ash, crude lipids, protein and fibre) of the dried crude hempseed meal used in this study

Constituent	Concentration (g kg^−1^)
Moisture	94.6 ± 1.6
Ash	76.1 ± 0.0
Crude lipids	61.3 ± 7.7
Protein	259.0 ± 0.0
Fibre	340.1 ± 6.4

### Protein recovery with the four experimental solvents

**Figure 1:**
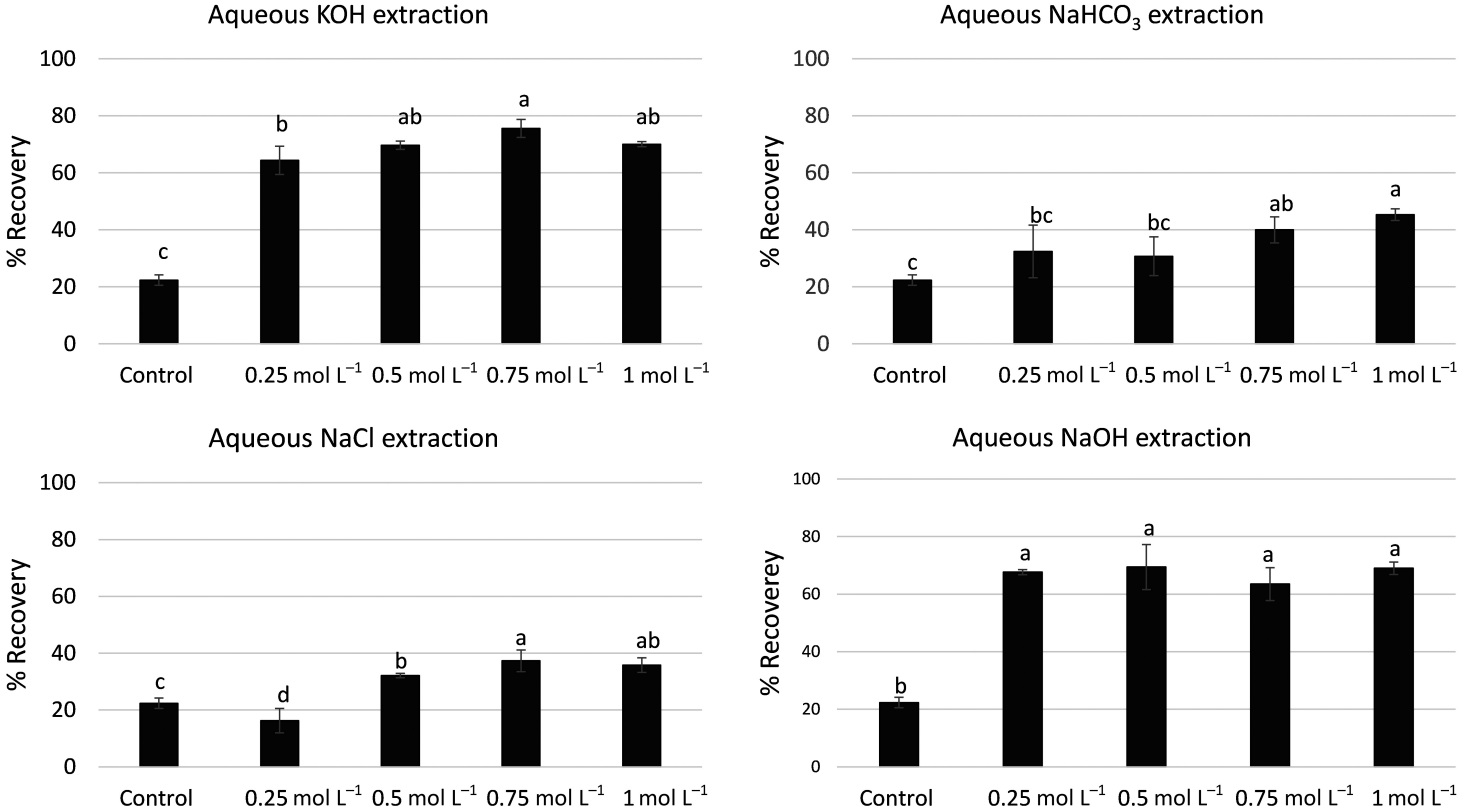
Hemp meal protein recovery (%) with aqueous KOH, NaHCO_3_, NaCl or NaOH at four different concentrations (0.25, 0.5, 0.75, 1.0 mol L^−1^). Mean values that do not share the same letter are statistically different (*P* < 0.05) by Tukey's test.

The control experiments showed that only 22 ± 2% of protein could be extracted from the hempseed cake when pure water was used as an extraction solvent. On the other hand, protein recovery increased significantly (two‐ to fivefold) when aqueous alkali (NaOH, KOH or NaHCO_3_) or salt (NaCl) solution was used as an extraction solvent (Fig. 1). Overall, aqueous KOH and NaOH solutions provided the highest protein recovery (60–78%), followed by aqueous NaHCO_3_ and NaCl solutions (16–45%). The concentration of KOH and NaHCO_3_ in the extraction medium had a minor but significant influence on protein recovery, while the concentration of NaOH showed no significant effect. On the other hand, the recovery doubled (16 ± 4% *vs*. 37 ± 4%) when the concentration of NaCl increased from 0.25 to 0.75 mol L^−1^. In the case of alkaline extraction, protein recovery was always higher than that obtained with control extraction, irrespective of the concentration of the alkali in the extraction medium. A similar trend was observed with salt extractions; however, use of 0.25 mol L^−1^ NaCl resulted in a slightly lower recovery of proteins than the control extraction.

Solid–liquid extraction of proteins from plant matrices is dependent on two major factors: (i) solubility of protein in the extraction medium and (ii) mass transfer of intracellular proteins into the bulk of the extraction medium. The solubility of protein is dependent on several extrinsic (e.g., temperature, pH, ionic strength of the extraction medium) and intrinsic factors (e.g., amino acid composition and isoelectric point). Among these, the interplay between the pH/ionic strength of the extraction medium and the pI of the protein play a critical role in solid–liquid extraction. Proteins display the lowest solubility when the pH of the extraction medium is equal to the pI of the protein, while solubility increases when pH >> pI or pH << pI. The crude proteins in the hemp meal are a mixture of edestin (albumin) and globulins, where the former accounts for 60–80% of the total composition.^2^ The pI of hemp meal proteins is in the range of 4–5,^15^ which could be the reason behind the lowest recovery of proteins in control extraction and a higher recovery in alkaline extraction medium. A recent study has also reported that pH > 9 will enhance the protein recovery from hempseed meal.^16^ In the present study, three different alkali salts were investigated with the aim of improving protein yield. KOH and NaOH as extraction solvents resulted in similar recovery of protein; however, aqueous NaHCO_3_ resulted in lower recovery values at all tested concentrations. This indicates that the relative strength of base (OH^−^ *vs*. HCO^−^_3_/CO_3_^2−^) plays a major role in the extraction of proteins. Since NaOH is less expensive than KOH, it may be an ideal extraction solvent. However, a detailed investigation on the purity of proteins obtained in these solvent systems is required. In the present study, alkaline extractions were also compared with salt extraction using aqueous NaCl at different molar concentrations. Overall, salt extraction resulted in lower protein recovery than alkaline extraction, but an increase in protein recovery with increasing concentration of NaCl was observed. This observation is attributed to the salting‐in effect, wherein ion salts at particular range of ionic strength decrease electrostatic protein–protein interaction and improve protein hydration, which ultimately improves protein solubility. At ionic strengths beyond a critical threshold, solubility may decrease due to salting‐out effects,^17^ although this is rarely observed with NaCl. Temperature can be increased to improve the solubility and mass transfer of proteins, but high temperature usually triggers unwanted protein modifications (e.g., the Maillard reaction, protein oxidation and protein crosslinking);^18^ therefore, extraction was carried out at the room temperature in the present study.

### Amino acid composition

**Figure 2:**
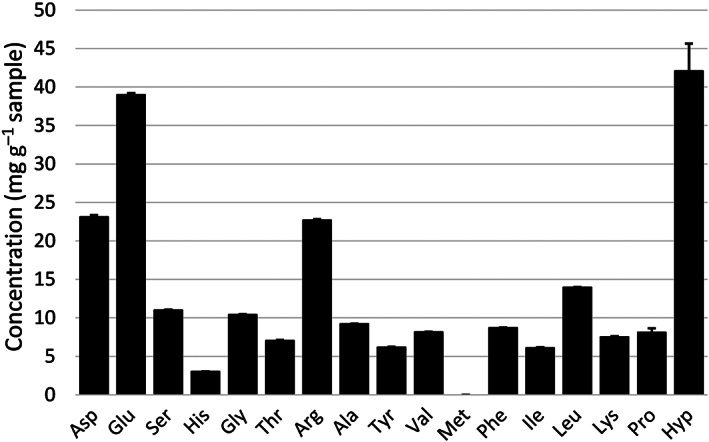
Amino acid composition of raw hempseed meal. Asp and Glu are represented as the sum of Asp and Asn, and Glu and Gln, respectively.

The hempseed meal contained 226.5 ± 3.24 mg g^−1^ amino acids after protein hydrolysis, of which 54.5 ± 0.19 (24%), 47.4 ± 0.43 (21%) and 124.5 ± 3.00 mg g^−1^ (55%) corresponded to total essential (EAA), total conditionally essential (CEAA) and total non‐essential amino acids (NEAA), respectively (see Fig. 2 for amino acid profile). The amount of Trp and Cys in the sample could not be determined as they were destroyed during the hydrolysis step. The amino acid composition determined in the present study was similar to that reported in previous literature.^19^

**Figure 3:**
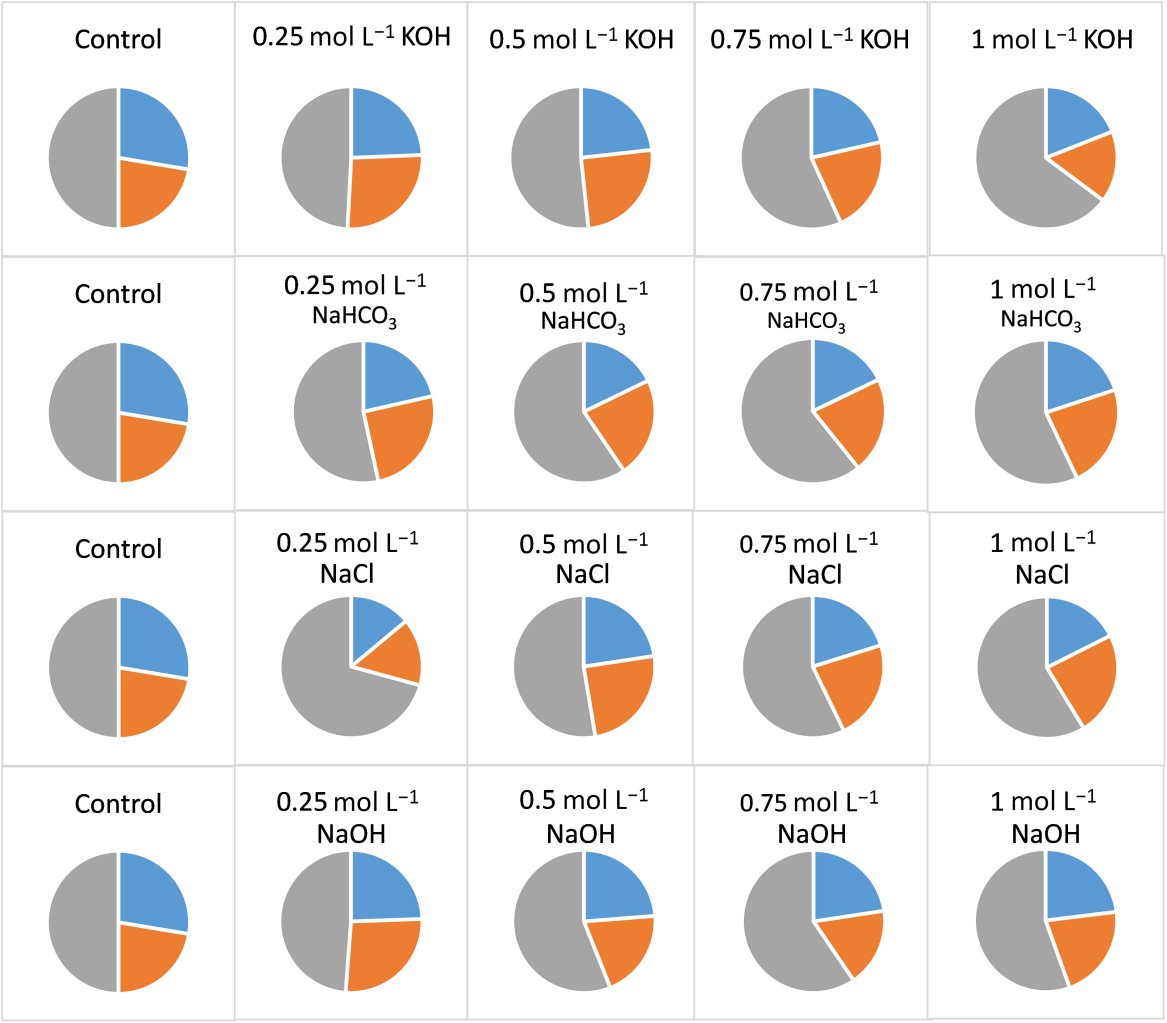
Pie chart showing relative proportions of essential (blue slice), conditionally essential (orange slice) and non‐essential amino acids (grey slice) in hempseed extracts obtained after extraction with water (control), KOH, NaHCO_3_, NaCl and NaOH.

Hempseed cake contains a mixture of proteins with variable molecular weight and structural properties. Therefore, the selectivity in the extraction of different proteins may vary depending upon the extraction solvent used. From a nutritional perspective, it is important to extract protein fractions rich in EAA and CEAA. However, only limited information is available on the nutritional quality of proteins extracted under different extraction solvents. In the present study, the composition of EAA, CEAA and NEAA of the extracted proteins was determined for all the hemp meal extracts among the different extraction solvents tested. It was clear that the use of water as an extraction solvent enabled the highest recovery of protein fractions rich in EAA (28%) and CEAA (22%), although it resulted in the lowest protein recovery (Fig. 1). Although alkali and salt extraction resulted in a better protein recovery, the extract contained relatively greater portions of NEAA compared to EAA and CEAA. Besides, higher concentrations of KOH, NaHCO_3_ and NaOH (>0.25 mol L^−1^) did not improve the recovery of EAAs (Fig. 3). This indicates that a lower concentration of alkali is required to extract protein fractions rich in EAAs, although higher concentration provides a better yield of proteins (Fig. 1 *vs*. Fig. 3). In the case of extraction using aqueous NaCl, the recovery of EAA increased with the concentration of NaCl up to 0.75 mol L^−1^ and then did not show significant improvement (Fig. 3). The variation in the relative proportion of EAA, CEAA and NEAA is attributed to selectivity in the extraction of proteins by the different solvents used, which is further linked to the charge and polarity of the amino acid residues. Furthermore, amino acid residues can undergo oxidation (e.g., Trp, Tyr, Met), elimination (e.g., β‐elimination of water and hydrogen sulfide from Ser and Cys, respectively, leading to the formation of dehydroalanine), and intra‐ or intermolecular crosslinking reactions (e.g., reaction between dehydroalanine and Lys or Cys); and these reactions are highly pH dependent,^20^, ^21^, ^22^, ^23^ causing different degrees of modification in different extraction solvents.

**Figure 4:**
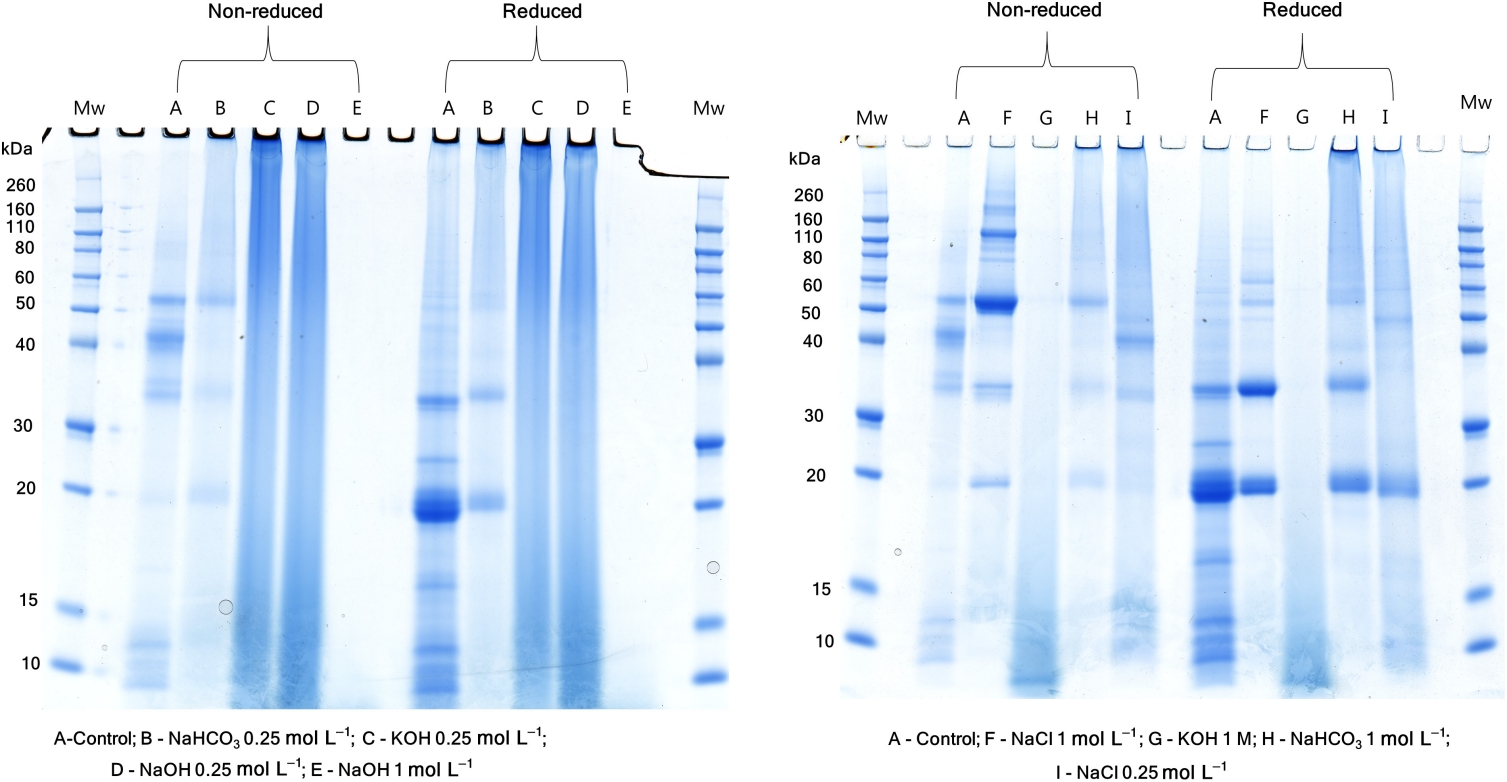
SDS‐PAGE of extracts containing hempseed proteins under reduced (dithiothreitol was used as a reducing agent) and non‐reduced conditions.

SDS‐PAGE was carried out to examine the protein distribution in the extract and to compare the protein profile obtained in different extraction solvents (Fig. 4). It was evident that extraction solvent had a significant influence on the selectivity of protein extracted, as protein band pattern varied depending on the type of solvent used in the extraction. In the control extract (non‐reduced), at least seven prominent bands around 53, 44, 35, 33, 12, 10 and 8 kDa were observed. The band around 53 and 35 kDa could be attributed to 1S (αβ) edestin monomers and acid α (11S α) edestin, respectively, while the fraction <18 kDa could be albumins.^16^ The bands at 53 and 44 kDa disappeared in samples reduced with DTT, while intense bands at 25 and 20 kDa were formed, indicating that the sample contained reducible protein crosslinks – probably disulfide bonds.

Unlike control extracts, the extract obtained using aqueous 1 mol L^−1^ NaCl displayed several high‐molecular‐weight protein bands with a molecular weight in the range of 80–260 kDa; these bands disappeared upon reduction of samples with DTT, indicating these are high‐molecular‐weight proteins/aggregates linked by disulfide bonds. A prominent band at 18 kDa was observed in extracts obtained in aqueous NaCl and NaHCO_3_, which could be an albumin fraction. A previous study has also reported the presence of a protein band at 18.4 kDa in the SDS‐PAGE gels of hempseed protein isolates.^15^ SDS‐PAGE of extracts obtained using aqueous NaOH and KOH (1 mol L^−1^) showed extensive crosslinking and gelation when samples were dissolved in SDS and it was not possible to load the samples into gels. In alkaline conditions, dehydroalanine formed from Ser and Cys residues (due to β‐elimination reactions as described above) reacts with Lys and Cys residues, forming crosslinked species lysinoalanine and lanthionine, respectively.^23^ These reactions could lead to high‐molecular‐weight protein aggregates. The presence of non‐reducible protein bands/smears in NaOH and KOH (0.25 mol L^−1^ each) extracts suggests the possible presence of these crosslinks. Further analysis of the alkali‐extracted samples is required to assess the quality and chemical modifications of proteins obtained in these extraction solvents.

## CONCLUSIONS

Alkaline solvent extraction of hempseed meal provided superior extraction yield compared to extraction using water or aqueous NaCl. However, extraction with water provided protein fractions rich in EAAs, indicating that water is an ideal solvent for extracting proteins from a nutritional perspective, wherein protein yield must be compromised. The use of higher concentrations of alkali or salt (0.25–1 mol L^−1^) in the extraction medium resulted in extracts containing increased level of proteins containing non‐essential amino acids. In addition, extraction in alkali solvents appeared to cause protein crosslinking, but this should be investigated in more detail in the future. Aqueous NaCl was found to be a suitable solvent to extract high‐molecular‐weight proteins. Future investigations must be focused on improving the extraction yield in water by applying non‐thermal biomass disruption techniques such as ultrasound‐assisted extraction or high‐pressure homogenization.

## CONFLICT OF INTEREST

The authors declare no conflict of interest.
